# Elevated alpha-fetoprotein in asymptomatic adults: Clinical features, outcome, and association with body composition

**DOI:** 10.1371/journal.pone.0271407

**Published:** 2022-07-21

**Authors:** Sangmi Jang, Gwang Hyeon Choi, Won Chang, Eun Sun Jang, Jin-Wook Kim, Sook-Hyang Jeong

**Affiliations:** 1 Department of Internal Medicine Seoul National University College of Medicine, Seoul National University Bundang Hospital, Seongnam, South Korea; 2 Department of Radiology, Seoul National University College of Medicine, Seoul National University Bundang Hospital, Seongnam, South Korea; Tokyo University of Agriculture, JAPAN

## Abstract

**Background and aim:**

Apparently healthy individuals with elevated serum alpha-fetoprotein (AFP) levels (>7 ng/mL) for unknown causes visit clinics. We investigated their clinical characteristics, outcomes, and relationship with body fat deposition and muscle mass.

**Methods:**

The case group included asymptomatic 137 individuals with “elevated AFP level” (R772) diagnostic code from 2009 to 2018 in a tertiary hospital. The control group enrolled 274 age- and sex-matched patients with <5 cm hepatic hemangiomas. Hepatic, visceral, and psoas muscle adiposity and psoas muscle index (PMI) were measured in the subgroups of 45 cases and 90 controls with pre-contrast computed tomography (CT) images.

**Results:**

The case group (mean age 47.5 years, male 35.8%) showed higher AFP levels (10.3 vs 2.5 ng/mL, p<0.001) and total bilirubin (0.8 vs 0.7 mg/dL, p<0.001), but a lower body mass index (22.2 vs 23.3 kg/m^2^, p = 0.011) and alanine aminotransferase levels (17.0 vs 19.0 IU/L, p = 0.047) than the controls. During 13 months of median follow-up, there was no cancer or liver disease development. The AFP levels were stable. In the subgroups with CT images, cases showed a lower proportion of hepatic steatosis (4.4% vs 18.9%, p = 0.023), higher psoas muscle attenuation (48.2 vs 43.8 Hounsfield units, p<0.001) and higher PMI (5.7 vs 4.2 cm^2^/m^2^, p<0.001) than the controls.

**Conclusion:**

Elevated AFP levels in asymptomatic individuals may play a role in expressing a protective phenotype against hepatic steatosis, myosteatosis, and sarcopenia. AFP levels in patients with elevated AFP were stable during follow-up without liver injury or cancer development. Interaction between AFP expression and steatosis warrants further study.

## Introduction

Alpha-fetoprotein (AFP) is a glycoprotein synthesized and secreted by the fetal liver and yolk sac [1]. The serum AFP level becomes elevated during embryogenesis, reaching up to 12–363 ng/mL at birth [2], but rapidly decreases after birth and is maintained at a low level, mostly less than 3 ng/mL throughout life in normal adults [3]. AFP is a well-known biomarker of hepatocellular carcinoma (HCC) and germ cell tumors [4, 5] In addition, it can often increase in acute or chronic liver injury and regeneration [6–9], and rarely in stomach, pancreas, and colon cancers.

Therefore, an elevated serum AFP level in adults is considered abnormal, as indicated by the presence of the International Classification of Diseases, 10^th^ Revision (ICD-10) diagnostic code of R772. However, seemingly healthy individuals but known to have elevated serum AFP levels on health checkups >7 ng/mL, a widely used cutoff [10, 11], were occasionally encountered in clinics. Many worried about the possibility of undetected cancer, and physicians performed several tests including abdominopelvic imaging such as computed tomography (CT) or ultrasonography, endoscopy, and urological or gynecological examinations to exclude malignancy. Even if the test results were normal, most physicians recommended AFP follow-up after 6 to 12 months as a precaution.

However, the cause, clinical characteristics, and outcomes of this mild AFP elevation up to 200 ng/mL in asymptomatic individuals are not known, except in rare cases of a mutation in the AFP promoter gene [12]. A few studies have suggested a positive association between AFP level and hepatic steatosis or metabolic syndrome [13, 14]. However, most patients with elevated AFP levels did not have fatty liver disease, to our impression. Thus, we aimed to investigate the clinical characteristics of patients with an elevated AFP level >7 ng/mL and its relationship with body composition in terms of hepatic steatosis, visceral adiposity, myosteatosis, and muscle mass. In addition, we longitudinally evaluated AFP levels over time and observed their clinical outcomes.

## Materials and methods

### Study population

From January 2009 to December 2018, a total of 195 patients with a diagnostic code of “elevated AFP level” (R772) were retrieved from the Clinical Data Warehouse at Seoul National University Bundang Hospital. Patients with any malignancy (n = 19), ovarian teratoma (n = 1), liver cirrhosis (n = 13), chronic hepatitis B (n = 12) or C (n = 2), and patients with elevated serum aspartate aminotransferase (AST) or alanine aminotransferase (ALT) levels above the upper normal limit of 40 IU/mL (n = 11) were excluded. None of the patients were pregnant, and none had a significant dosage of drug administration related to fatty liver, including amiodarone, valproate, tamoxifen, estrogens, and corticosteroids. The remaining 137 patients were included as the case group. All of them had abdominopelvic imaging, however, pre-contrast CT images adequate to measure hepatic, visceral, subcutaneous, and muscle fat attenuation were available in 45 patients. To analyze adiposity, only the non-enhanced CT images should be used because CT images enhanced with contrast media do not follow the same attenuation reference.

**Fig 1:**
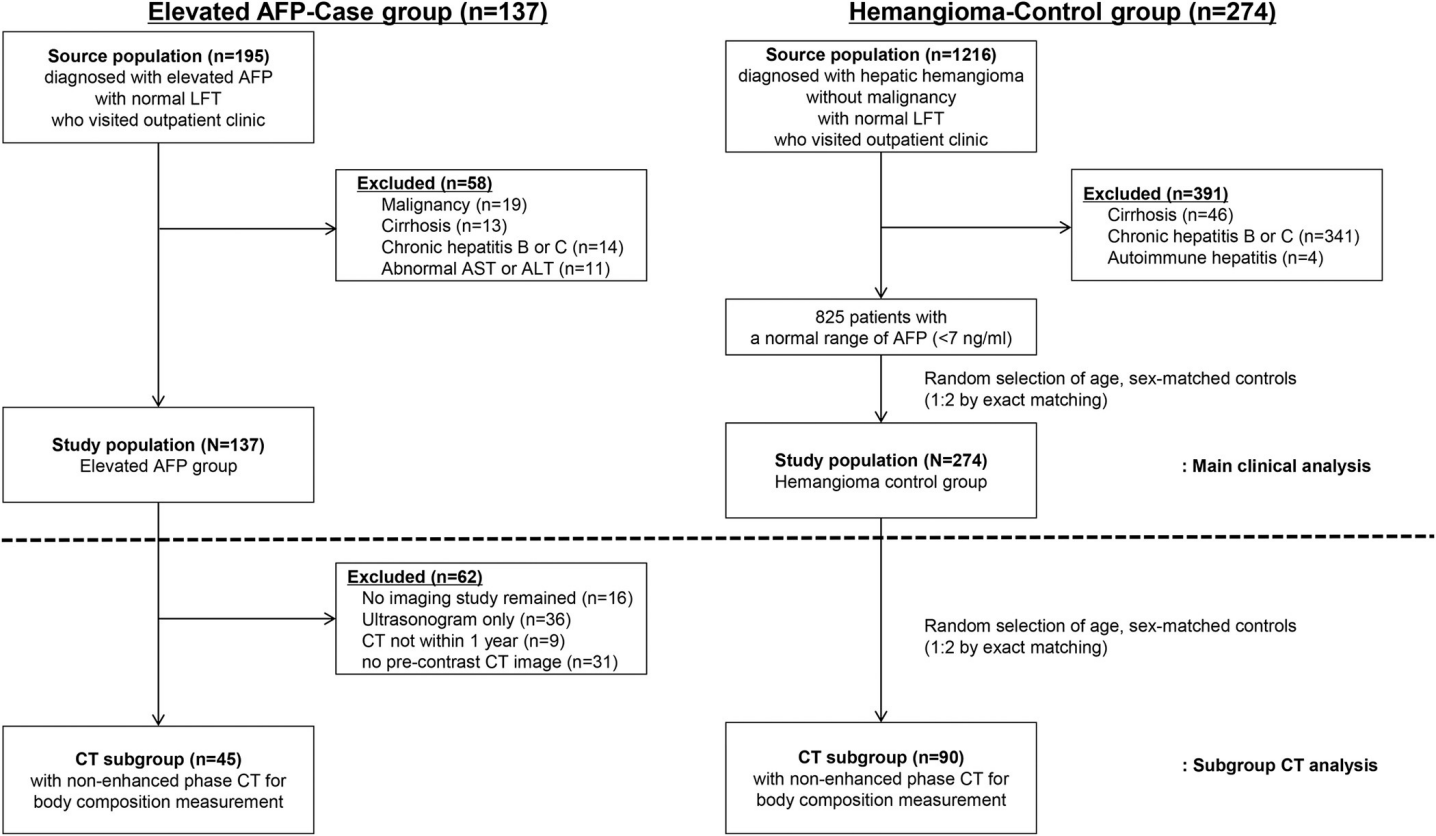
Patient selection flow gram. A total of 137 patients with an elevated AFP level without any malignancy, ovarian teratoma, liver cirrhosis, chronic hepatitis, or elevated serum aspartate aminotransferase or alanine aminotransferase level above the upper normal limit of 40 IU/mL were compared with the hemangioma control group who had normal serum AFP levels. To analyze adiposity, 45 patients with non-enhanced CT images in the elevated AFP level group were compared to 90 patients with non-enhanced CT images in the hemangioma control group.

As a control group, we chose patients diagnosed with hepatic hemangioma (ICD-10 code D1802) <5 cm (median diameter, 1.4 cm; interquartile range [IQR], 0.80–2.00 cm) and normal laboratory results at the same hospital during the same period as for the case group, because most of them had abdominal imaging and AFP levels, and were healthy, while normal subjects rarely underwent CT. Among them, 274 subjects were randomly assigned by age and sex matching. For the measurement of body composition, 90 age- and sex-matched control subjects with adequate pre-contrast CT were randomly selected. The patient enrollment flowchart is shown in Fig 1.

This study was conducted according to ethical guidelines of the Helsinki declaration after the approval of the institutional review board (IRB) at Seoul National University Bundang Hospital (IRB No. B-2010-642-102). Informed consent was waived by the IRB due to the retrospective nature of the study.

### Data collection

Clinical and laboratory data were collected by a thorough review of the medical records. Hypertension was defined as systolic blood pressure (BP) >140 mmHg, diastolic BP >90 mmHg, or a prescription history of antihypertensive medication. Diabetes was defined as a fasting glucose level >126 mg/dL, random glucose level >200 mg/dL, or a prescription history of anti-diabetic medication. Hyperlipidemia was defined as a total cholesterol level ≥200 mg/dL [15] or a history of anti-hyperlipidemic medication. Significant alcohol consumption was defined as 20 g/day for women and 30 g/day for men [16].

The laboratory results of AFP, platelet, albumin, total bilirubin, alkaline phosphatase (ALP), AST, ALT, glucose, and total cholesterol levels were collected. AFP levels were standardized in units by converting IU/mL to ng/mL for the results presented as IU/mL according to different methods.

For clinical indices, hepatic steatosis index (HSI), nonalcoholic fatty liver disease (NAFLD) fibrosis score (NFS), and Fibrosis-4 index (FIB-4) were calculated. HSI was calculated as 8 × (ALT/AST) + BMI (+ 2 if type 2 diabetes, + 2 if female). An HSI score above 36 was considered indicative of hepatic steatosis. NFS was calculated as -1.675 + 0.037 × age + 0.094 × BMI + 1.13 × diabetes + 0.99 × (AST/ALT) - 0.013 × platelet—0.66 × albumin. An NFS score <-1.455 was considered to indicate a low probability of hepatic fibrosis, >-1.455 to <0.676 intermediate probability, and >0.676 high probability of fibrosis. FIB-4 was calculated as (age × AST)/(platelet × √ALT) with a low cutoff value of <1.3 and a high cutoff of >2.67 for advanced fibrosis.

### CT analyses of body adiposity and muscle mass

Non-enhanced CT images were analyzed using Rapidia software program (Windows version 2.8; INFINITT, Seoul, Korea). Hepatic attenuation was measured by calculating the mean liver Hounsfield unit (HU) of a region of interest of a 1.5 cm^2^-sized circular area in 8 liver segments. To measure psoas muscle HU and psoas muscle mass, a contour of the psoas muscle area was defined at the third lumbar vertebral level (L3) with visible pedicles using Rapidia software. All CT images were measured by two technicians who were blinded to the clinical data, and randomly selected 20 images were cross-checked for validation.

The psoas muscle index (PMI) was calculated as (right + left psoas muscle area, cm^2^/ height, m^2^) at the L3 level. Visceral adipose tissue area (VAA) and subcutaneous adipose tissue area (SAA) were measured by drawing a contour along the visceral and subcutaneous fat using Rapidia software. The reference HU for skeletal muscle was -29~+150 HU, for visceral fat, -150~-50 HU, and for subcutaneous fat, -190~-30 HU, according to the widely-used reference [17, 18].

To assess hepatic steatosis, hepatic attenuation level minus splenic attenuation, indicated as mean HU, was calculated on CT image (CT_L-S_). However, CT images were acquired using two different types of CT tubes with peak kilovoltage (kVp), 100 kVp (n = 102), and 120 kVp (n = 33) at our center, thus they were separately analyzed due to different cut-off values for CT_L-S_ according to CT tube voltage. The previously reported cutoffs for hepatic steatosis were as follows [19]: for 100 kVp, CT_L-S_ HU >1.6, HU 1.6~-3.9, and HU <-3.9 were considered to correspond with no, mild, and moderate to severe hepatic steatosis, respectively. For 120 kVp, CT_L-S_ HU >7.6, HU 7.6~-2.1, and HU <-2.1 were considered to correspond with no, mild, and moderate to severe hepatic steatosis, respectively.

### Statistical analysis

Various clinical, laboratory, and CT imaging variables are shown as mean ± standard deviation (SD) or median (interquartile range, IQR), according to normality and distribution of data. Student’s t-test or Mann-Whitney test was used to analyze continuous variables, and the chi-square test or Fisher’s exact test was used to analyze categorical variables. Paired t-test was performed to compare the baseline and at 1, 3, 6, 12, 24, and >24 months, and a spaghetti plot was created to visualize longitudinal changes in AFP levels in the case group.

The hemangioma-control group was randomly extracted through age- and sex-exact matching with the elevated AFP-case group. Univariate and multivariate logistic regression analyses were performed to identify factors related to elevated AFP levels. Statistical significance was set at p<0.05. To investigate whether liver steatosis is a factor associated with an increase in AFP, multivariate logistic analysis was performed using CT_L-S_ as a continuous variable (model 1) and the presence of hepatic steatosis as a binary variable (model 2).

Statistical analyses were performed using SPSS software (Windows version 25.0; SPSS Inc., Chicago, IL, USA) and GraphPad Prism software (Windows version 5.0; GraphPad Software Inc., San Diego, CA, USA).

## Results

### Clinical characteristics of the case group with elevated AFP levels compared to the control group

The comparative results of clinical characteristics at diagnosis are summarized in Table 1. The median AFP level in the case group (n = 137, mean age 47.5 years, male 35.8%) was 10.3 ng/mL, while it was 2.5 ng/mL in the age- and sex-matched control group (n = 274). The case group showed a lower BMI (22.2 vs 23.3 kg/m^2^, p = 0.011) than controls, but a similar proportion of significant alcohol intake and comorbidities including hypertension, diabetes, and hyperlipidemia. The case group showed a higher total bilirubin level (0.8 vs 0.7 mg/dL, p<0.001) but a lower ALT level (17.0 vs 19.0 IU/L, p = 0.047) than the control group. The prevalence of Gilbert’s syndrome (clinically defined without genetic test) in the case group was not different from that in the control group (10.9% vs 8.0%, p = 0.428). Among the clinical indices, the case group showed a significantly higher proportion of low NFS grade (<-1.455), suggesting a lower probability of fibrosis than the control group (95.9% vs 87.9%, p = 0.025; Table 1).

**Table 1:** Baseline characteristics of the case group with elevated AFP in comparison with the control group.

	Total		Case group		Control group		p-value
Variables, No (%) / Median (IQR)	(n = 411)		(n = 137)		(n = 274)		
Age, mean ± SD, years	47.4 ± 11.4		47.5 ± 10.6		47.4 ± 11.8		0.963
Male	147 (35.8)		49 (35.8)		98 (35.8)		1.000
BMI, kg/m^2^	22.9 (20.9–24.9)	(n = 386)	22.2 (20.4–24.7)	(n = 125)	23.3 (21.2–25.2)	(n = 261)	0.011
Obesity (BMI ≥25), %	93 (24.1)		24 (19.2)		69 (26.4)		0.120
Overweight/Obesity (BMI ≥23), %	189 (49.0)		50 (40.0)		139 (53.3)		0.015
Significant alcohol consumption*, %	34 (9.7)	(n = 349)	10 (8.5)	(n = 118)	24 (10.4)	(n = 231)	0.568
Hypertension	109 (26.5)		29 (21.2)		80 (29.2)		0.082
Diabetes	27 (6.6)		6 (4.4)		21 (7.7)		0.205
Hyperlipidemia	183 (44.5)		63 (46.0)		120 (43.8)		0.674
AFP, ng/dL median (IQR)	3.5 (2.2–8.7)		10.3 (8.7–13.7)		2.5 (1.8–3.5)		<0.001
Range, min—max	0.6–168.9		7.1–168.9		0.6–6.8		
WBC, x10^3^/μL	5.7 (4.7–6.7)	(n = 366)	5.5 (4.7–6.6)	(n = 108)	5.7 (4.7–6.8)	(n = 258)	0.583
Hemoglobin, g/dL	13.9 (13.1–14.9)	(n = 368)	14.1 (13.1–15.1)	(n = 108)	13.9 (13.1–14.9)	(n = 260)	0.543
Platelets, x10^9^/μL	257.5 (226.0–291.8)	(n = 368)	259.0 (221.5–292.5)	(n = 109)	255.0 (226.0–291.0)	(n = 259)	0.937
Albumin, g/dL	4.5 (4.3–4.6)	(n = 389)	4.5 (4.3–4.7)	(n = 120)	4.5 (4.3–4.6)	(n = 269)	0.932
Total bilirubin, mg/dL	0.7 (0.5–0.9)	(n = 392)	0.8 (0.6–1.0)	(n = 122)	0.7 (0.4–0.9)	(n = 270)	<0.001
ALP, IU/L	66.0 (55.0–79.0)	(n = 388)	67.0 (57.0–79.0)	(n = 118)	66.0 (53.8–79.0)	(n = 270)	0.368
AST, IU/L	22.0 (18.0–25.0)	(n = 394)	21.0 (18.0–25.0)	(n = 123)	22.0 (18.0–26.0)	(n = 271)	0.536
ALT, IU/L	18.0 (14.0–25.0)	(n = 394)	17.0 (14.0–21.0)	(n = 123)	19.0 (14.0–26.0)	(n = 271)	0.047
γGT, IU/L	19.0 (14.0–30.0)	(n = 338)	18.0 (13.0–28.5)	(n = 101)	19.0 (14.0–30.0)	(n = 237)	0.556
Glucose, mg/dL	95.0 (88.0–102.0)	(n = 369)	95.0 (87.3–102.0)	(n = 112)	95.0 (89.0–102.5)	(n = 257)	0.602
Total cholesterol, mg/dL	191.0 (168.0–212.0)	(n = 394)	194.5 (171.8–210.0)	(n = 122)	188.5 (166.3–212.0)	(n = 272)	0.289
Hepatic steatosis index (HSI)	34.2 (32.3–37.0)	(n = 373)	34.1 (31.9–36.9)	(n = 114)	34.3 (32.3–37.1)	(n = 259)	0.665
HSI >36	127 (34.0)		37 (32.5)		90 (34.7)		0.667
NAFLD fibrosis score (NFS)	-2.8 (-3.5 - -2.1)	(n = 344)	-2.9 (-3.4 - -2.1)	(n = 97)	-2.7 (-3.5 - -2.0)	(n = 247)	0.847
NFS grade							0.025
<-1.455	310 (90.1)		93 (95.9)		217 (87.9)		
-1.455–0.676	34 (9.9)		4 (4.1)		30 (12.1)		
>0.676	0 (0.0)		0 (0.0)		0 (0.0)		
Fibrosis-4 index (FIB-4)	0.9 (0.7–1.2)	(n = 365)	1.0 (0.7–1.3)	(n = 107)	0.9 (0.7–1.2)	(n = 258)	0.170
FIB-4 grade							0.511
<1.3	292 (80.0)		83 (77.6)		209 (81.0)		
1.3–2.67	71 (19.5)		24 (22.4)		47 (18.2)		
>2.67	2 (0.5)		0 (0.0)		2 (0.8)		

Numerical variables are shown as median (IQR) except age, shown as mean ± SD. Categorical variables are shown as n (%).

*Significant alcohol consumption: >30 g/day in male and >20 g/day in female.

### Univariate and multivariate logistic regression analyses for factors related to elevated AFP level

The results of univariate and multivariate regression analyses for the factors associated with elevated AFP levels are shown in Table 2. In the univariate model, BMI ≥23 kg/m^2^ (odds ratio [OR] 0.585, 95% confidence interval [CI]: 0.380–0.902, p = 0.015) and high total bilirubin (OR 3.151, 95% CI:1.800–5.516, p<0.001) were significantly associated with elevated AFP levels. Multivariate analysis confirmed that BMI ≥23 kg/m^2^ (OR 0.551, 95% CI:0.346–0.876, p<0.05) and total bilirubin level (OR 3.396, 95% CI:1.885–6.120, p<0.001) were independent factors for elevated AFP.

**Table 2:** Variables associated with elevated AFP >7 ng/mL.

	Univariate analysis		Multivariate analysis	
**Variables**	**OR (95% CI)**	**p-value**	**OR (95% CI)**	**p-value**
Overweight/Obesity (BMI ≥23 kg/m^2^)	0.585 (0.380–0.902)	0.015	0.551 (0.346–0.876)	0.012
Hypertension	0.651 (0.401–1.058)	0.083		
WBC, x10^3^/μL	0.963 (0.832–1.115)	0.616		
Hemoglobin, g/dL	1.043 (0.899–1.210)	0.576		
Platelets, x10^9^/μL	1.000 (0.996–1.004)	0.943		
Albumin, g/dL	0.938 (0.427–2.057)	0.873		
Total bilirubin, mg/dL	3.151 (1.800–5.516)	<0.001	3.396 (1.885–6.120)	<0.001
ALP, IU/L	1.002 (0.992–1.013)	0.664		
AST, IU/L	0.990 (0.952–1.029)	0.601		
ALT, IU/L	0.973 (0.946–1.001)	0.061		
γGT, IU/L	0.996 (0.984–1.009)	0.591		
Glucose, mg/dL	0.993 (0.978–1.008)	0.338		
Total cholesterol, mg/dL	1.003 (0.997–1.010)	0.278		
FIB-4 score	1.149 (0.679–1.946)	0.604		
NFS	0.949 (0.758–1.187)	0.645		
HSI	0.973 (0.917–1.032)	0.354		

### Longitudinal analysis

**Fig 2:**
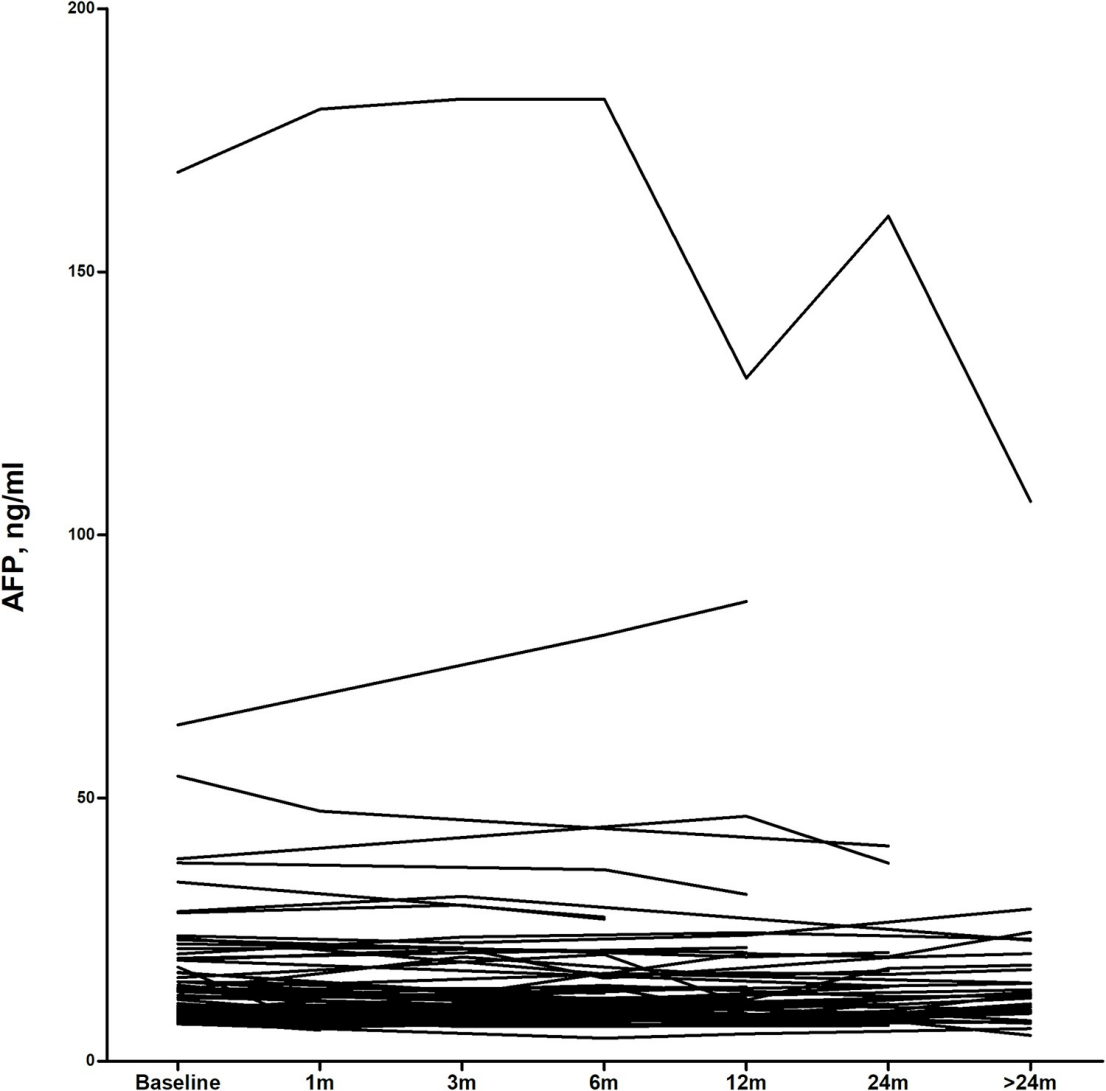
Longitudinal follow-up of AFP in the case group. The Median follow-up period was 12.9 months. No patients had newly developed malignancy or liver cirrhosis. AFP level stayed stable and there was no significant change over time among patients during follow-up.

Among the 137 cases, 92 patients were tested for AFP more than once. During a median follow-up of 12.9 months, most patients had stable AFP levels throughout the follow-up period. Among 13 (14.1%) patients showing ≥25% change in AFP level, 5 patients and 8 patients showed a slowly increasing and decreasing trend, respectively (Fig 2). There were no cases of development of cancer or liver disease during the follow-up.

Similarly, among the control group of patients with hemangioma, 73 patients were tested more than twice for AFP. These patients were tested a median 2.0 (IQR 1.5–4.5) times, and the time interval between the baseline AFP test and the first follow-up was median of 16.3 months. The longitudinal follow-up AFP in patients with hemangioma was not significantly changed over time (p = 0.148).

### Comparison of body compositions using CT images between the cases and the controls: A subgroup analysis

Of the total case group of 137 patients, most patients had imaging studies done (n = 122, 89.1%). 81 patients (59.1%) had CT images but pre-enhanced CT images suitable for assessment of liver and muscle fat analysis were available in only 45 patients. Among the 92 patients without available pre-enhanced CT images, abdominal ultrasonography was performed in 40 patients (29.2%), abdominal MRI in 1 patient, and ultrasonography or CT report was recorded in the chart without imaging loaded in 5 patients. Because non-contrast CT image is only available in half of the cases, clinical characteristics in the CT available cases (n = 45) and CT-unavailable cases (n = 90) were compared. There was no significant difference in baseline characteristics between these 2 groups (S1 Table).

The results of subgroup analysis of the body composition between the case and control groups are summarized in Table 3. The case subgroup showed a higher total bilirubin level (0.7 vs 0.6 mg/dL, p = 0.027), but a lower BMI (22.6 vs 22.9 kg/m^2^, p = 0.391) and ALT level (17.5 vs 19.0 IU/L, p = 0.476) than the age- and sex-matched control subgroups.

**Table 3:** Baseline characteristics of the case group in comparison with the control group with CT—a cross-sectional study.

	Total		Case group		Control group		p-value
**Variables**	**(n = 135)**		**(n = 45)**		**(n = 90)**		
Age, mean ± SD, years	48.4 ± 12.4		48.3 ± 11.3		48.4 ± 12.9		0.992
Sex, male	62 (45.9)		21 (46.7)		41 (45.6)		0.903
BMI, kg/m^2^	22.8 (21.2–24.9)	(n = 131)	22.6 (20.9–24.3)	(n = 42)	22.9 (21.3–25.2)	(n = 89)	0.391
Obesity (BMI ≥25), %	29 (22.1)		6 (14.3)		23 (25.8)		0.137
Overweight/Obesity (BMI ≥23), %	61 (46.6)		19 (45.2)		42 (47.2)		0.834
Significant drinking*	13 (11.3)	(n = 115)	4 (10.3)	(n = 39)	9 (11.8)	(n = 76)	1.000
Alcohol intake, g/day	7.7 (2.1–24.0)	(n = 67)	3.2 (1.7–16.0)	(n = 23)	11.1 (2.6–24.0)	(n = 44)	0.237
Hypertension	42 (31.1)		12 (26.7)		30 (33.3)		0.430
Diabetes	12 (8.9)		3 (6.7)		9 (10.0)		0.750
Hyperlipidemia	60 (44.4)		20 (44.4)		40 (44.4)		1.000
AFP, ng/dL	3.7 (2.2–9.6)		10.9 (9.5–14.0)		2.7 (1.7–3.7)		<0.001
WBC, x10^3^/μL	5.8 (4.9–6.7)	(n = 116)	5.8 (4.7–6.8)	(n = 34)	5.9 (5.0–6.7)	(n = 82)	0.937
Hemoglobin, g/dL	14.1 (13.2–15.2)	(n = 116)	14.2 (13.4–15.3)	(n = 34)	14.0 (13.2–15.1)	(n = 82)	0.420
Platelets, x10^9^/μL	252.5 (218.3–285.8)	(n = 116)	237.5 (216.8–288.8)	(n = 34)	256.0 (217.5–285.3)	(n = 82)	0.776
Albumin, g/dL	4.5 (4.3–4.6)	(n = 130)	4.5 (4.2–4.7)	(n = 41)	4.5 (4.4–4.6)	(n = 89)	0.373
Total bilirubin, mg/dL	0.7 (0.4–0.9)	(n = 131)	0.7 (0.6–0.9)	(n = 42)	0.6 (0.3–0.9)	(n = 89)	0.027
ALP, IU/L	68.0 (57.0–82.0)	(n = 129)	66.5 (55.5–80.5)	(n = 40)	68.0 (57.0–82.5)	(n = 89)	0.381
AST, IU/L	22.0 (19.0–27.0)	(n = 131)	21.0 (19.0–24.3)	(n = 42)	23.0 (19.0–27.0)	(n = 89)	0.145
ALT, IU/L	19.0 (14.0–26.0)	(n = 131)	17.5 (14.0–21.5)	(n = 42)	19.0 (13.0–26.0)	(n = 89)	0.476
γGT, IU/L	19.0 (14.0–26.0)	(n = 113)	19.0 (13.0–24.0)	(n = 35)	18.0 (14.0–26.5)	(n = 78)	0.593
Glucose, mg/dL	95.5 (88.3–103.8)	(n = 124)	95.0 (86.0–105.0)	(n = 37)	96.0 (89.0–104.0)	(n = 87)	0.515
Total cholesterol, mg/dL	191.0 (168.0–210.0)	(n = 131)	188.5 (166.5–208.0)	(n = 42)	191.0 (168.0–214.0)	(n = 89)	0.620
Hepatic steatosis index (HSI)	34.0 (32.1–37.1)	(n = 128)	33.9 (31.5–36.8)	(n = 40)	34.3 (32.2–37.4)	(n = 88)	0.379
HSI <30	12 (9.4)		6 (15.0)		6 (6.8)		0.190
HSI >36	42 (32.8)		12 (30.0)		30 (34.1)		0.648
NAFLD fibrosis score (NFS)	-2.5 (-3.4 - -1.9)	(n = 113)	-2.6 (-3.3 - -1.9)	(n = 32)	-2.5 (-3.5 - -1.9)	(n = 81)	0.985
NFS grade							0.550
<-1.455	98 (86.7)		29 (90.6)		69 (85.2)		
-1.455–0.676	15 (13.3)		3 (9.4)		12 (14.8)		
>0.676	0 (0.0)		0 (0.0)		0 (0.0)		
Fibrosis-4 index (FIB-4)	1.0 (0.8–1.3)	(n = 115)	1.0 (0.8–1.3)	(n = 33)	1.0 (0.7–1.3)	(n = 82)	0.701
FIB-4 grade							1.000
<1.3	86 (74.8)		25 (75.8)		61 (74.4)		
1.3–2.67	27 (23.5)		8 (24.2)		19 (23.2)		
>2.67	2 (1.7)		0 (0.0)		2 (2.4)		
CT							
Liver							
Mean HU, liver	60.2 (55.5–65.2)		60.8 (57.4–64.6)		60.1 (54.5–65.3)		0.475
Mean HU, spleen	50.3 (46.9–53.8)		49.6 (46.5–52.2)		50.9 (47.1–54.1)		0.169
Mean HU, liver-spleen	10.2 (5.9–14.1)		11.1 (7.9–14.7)		9.9 (4.8–14.0)		0.076
Hepatic steatosis^†^							0.067
None	116 (85.9)		43 (95.6)		73 (81.1)		0.023
Steatosis	19 (14.1)		2 (4.4)		17 (18.9)		
Mild	13 (9.6)		2 (4.4)		11 (12.2)		
Moderate-severe	6 (4.4)		0 (0.0)		6 (6.7)		
Viscera & Subcutaneous tissue							
Visceral adiposity index, cm^2^/m^2^	28.6 (15.4–46.3)	(n = 127)	27.5 (16.2–40.4)	(n = 42)	30.2 (12.6–50.9)	(n = 85)	0.509
Subcutaneous adiposity index, cm^2^/m^2^	39.2 (31.0–50.7)	(n = 79)	40.2 (30.9–52.3)	(n = 31)	38.0 (31.1–50.0)	(n = 48)	0.616
Visceral to subcutaneous fat area ratio	0.6 (0.3–1.0)	(n = 82)	0.6 (0.3–0.9)	(n = 34)	0.6 (0.3–1.2)	(n = 48)	0.851
Muscle							
Mean HU, psoas muscle	45.3 (40.3–50.8)	(n = 131)	48.2 (42.4–56.7)		43.8 (38.5–47.1)	(n = 86)	<0.001
Psoas muscle index, cm^2^/m^2^	4.9 (3.5–6.3)	(n = 127)	5.7 (4.4–7.3)	(n = 42)	4.2 (3.0–5.8)	(n = 85)	<0.001

Numerical variables are shown as median (IQR) except age, shown as mean ± SD. Categorical variables are shown as n (%).

*Significant alcohol consumption: >30 g/day in male and >20 g/day in female.

^†^HU For kVp 100: none >1.6, mild -3.9~1.6, moderate to severe <-3.9; for kVp 120: none >-7.6, mild -2.1~-7.6, moderate to severe <-2.1.

The mean CT_L-S_ tended to be higher in cases than in controls (11.1 vs 9.9 HU, p = 0.076) suggesting less fat. Using the validated cutoff for hepatic steatosis on CT images, the proportion of hepatic steatosis in cases was significantly lower than that in controls (4.4% vs 18.9%, p = 0.023). Interestingly, the mean psoas muscle attenuation in cases was significantly higher than in controls (48.2 vs 43.8 HU, p<0.001), suggesting a less degree of myosteatosis.

Likewise, median PMI in cases was significantly higher than in controls (5.7 vs 4.2 cm^2^/m^2^, p<0.001), showing higher muscle mass in cases than in controls. However, neither VAI nor SAI were different between the two groups (Table 3).

### Univariate and multivariate analyses for body composition factor associated with elevated AFP level: A subgroup analysis

To confirm the association between body composition variables, including hepatic steatosis, myosteatosis, PMI, and elevated AFP level, univariate and multivariate analyses were performed, and the results are shown in Table 4.

**Table 4:** Variables associated with elevated AFP >7 ng/mL in patients with CT.

	Model 1				Model 2			
	Univariate analysis		Multivariate analysis		Univariate analysis		Multivariate analysis	
Variables	OR (95% CI)	p-value	OR (95% CI)	p-value	OR (95% CI)	p-value	OR (95% CI)	p-value
Overweight/Obesity (BMI ≥23 kg/m^2^)	0.924 (0.443–1.931)	0.834			0.924 (0.443–1.931)	0.834		
Liver								
Mean HU, liver	1.030 (0.976–1.087)	0.280			1.030 (0.976–1.087)	0.280		
Mean HU, liver-spleen	1.059 (1.000–1.121)	0.049	1.087 (1.018–1.161)	0.013				
Hepatic steatosis*					0.200 (0.044–0.907)	0.037	0.159 (0.031–0.814)	0.027
Viscera & Subcutaneous tissue								
Visceral adiposity index	1.000 (1.000–1.000)	0.290			1.000 (1.000–1.000)	0.290		
Subcutaneous adiposity index	1.000 (1.000–1.000)	0.512			1.000 (1.000–1.000)	0.512		
Visceral to subcutaneous fat area ratio	1.076 (0.422–2.744)	0.879			1.076 (0.422–2.744)	0.879		
Muscle								
Mean HU, psoas muscle	1.110 (1.051–1.172)	<0.001	1.099 (1.031–1.172)	0.004	1.110 (1.051–1.172)	<0.001	1.088 (1.021–1.158)	0.009
Psoas muscle index	1.003 (1.001–1.005)	0.002	1.002 (1.000–1.004)	0.065	1.003 (1.001–1.005)	0.002	1.002 (1.000–1.004)	0.063

*Hepatic steatosis degrees of mild & moderate-severe are analyzed.

Regardless of the mean CT_L-S_ as a continuous variable in model 1 or the presence of hepatic steatosis as a binary variable in model 2, mean CT_L-S_ (OR 1.087, 95% CI 1.018–1.161, p = 0.013) or absence of hepatic steatosis (OR 0.159, 95% CI 0.031–0.814, p = 0.027), and mean psoas muscle HU were independent factors for elevated AFP levels.

## Discussion

In our study, asymptomatic individuals with elevated AFP levels up to 168 ng/mL showed a higher serum total bilirubin level, lower ALT level, lower BMI, and lower probability of NAFLD-fibrosis than the age- and sex-matched control group. Total bilirubin level and BMI were independent factors for elevated AFP levels. During follow-up, there was no development of cancer or liver disease, and AFP levels were stable in most patients. In the subgroups with CT images, cases showed a lower proportion of hepatic steatosis and myosteatosis, with a higher psoas muscle mass, than the controls. Therefore, they showed a protective phenotype against fatty liver, myosteatosis, and sarcopenia.

In this study, the cases showed a higher total bilirubin level and lower ALT level than the controls, and the total bilirubin level was an independent factor for elevated AFP levels. The results were consistent even when patients with Gilbert’s syndrome were excluded (S2 Table). The proportion of Gilbert’s syndrome was not different between the case and the control groups, and was compatible with that in the general population [20, 21]. AFP can bind to bilirubin [22, 23], which has cytoprotective capacity by antioxidant function. Moreover, bilirubin binds to peroxisome proliferator-activated receptor-alpha, leading to reduced hepatic fat accumulation. Growing evidence has shown that hyperbilirubinemia in the general population and in Gilbert’s syndrome was negatively correlated with obesity, and the prevalence and progression of NAFLD [24–27]. Therefore, the interaction between AFP and bilirubin may be related to lower levels of ALT, as shown in our results. Interestingly, our case group showed a significantly lower BMI and a higher probability of absence of NAFLD-related liver fibrosis than the age- and sex-matched control group, suggesting a lower probability of NAFLD despite the similar prevalence of hypertension, diabetes, and dyslipidemia.

More patients in the case group took medication for dyslipidemia (16.1% vs 6.9%, p = 0.004) and health supplements (23.4% vs 13.9%, p = 0.016), which consisted of mainly multivitamins and ginseng than the control group (S3 Table). Although it may be possible that history taking was done more rigorously for patients with elevated AFP than controls, statin and herbal supplements may have affected serum AFP to rise.

Most patients had relatively stable AFP throughout follow-up, and no cases of cancer or liver disease developed during the follow-up. Therefore, reassurance of patients with yearly follow-up of AFP levels would be appropriate. However, 14% of the patients showed >25% change in AFP levels, which should be cautiously followed. Until now, the mechanism of the elevated AFP in seemingly healthy adults was not known. Cases of the hereditary persistence of AFP in adults related to AFP promoter mutations are very rare [12]. Our previous study on the 4 healthy individuals with elevated AFP (12.1–186.1 ng/mL) did not show mutations in the 5 transcription regulatory regions (1 promoter, 2 enhancers, and 2 silencers), and the family members of one subject showed a hereditary pattern [28], suggesting a genetic mechanism.

In 2009, Babali A. et al. reported that AFP levels increased as sonographically defined hepatic steatosis in 64 Turkish NAFLD patients [29]. However, in 2013, Kara M. et al. showed that AFP level of 103 patients with pathologically confirmed NAFLD was not different from that in the age- and sex-matched 57 healthy controls [30]. In 2014, Xu et al. reported that 2,601 health-check examinees with fatty liver on ultrasound showed a higher median serum AFP level (3.10 ng/mL) than the no fatty liver group (n = 7,199, 2.90 ng/mL), but AFP level was not an independent factor for fatty liver in the multivariable analysis [13]. In 2016, Chen Y. et al. reported that AFP level (2.7 ng/mL) of 1,512 health-check examinees with metabolic syndrome was higher than that (2.4 ng/mL) of 6,243 subjects without metabolic syndrome [14]. Therefore, the association between AFP levels and fatty liver or metabolic syndrome is unclear. In our CT subgroup, the cases showed consistently higher bilirubin levels, lower proportions of hepatic steatosis and myosteatosis, and higher muscle mass than the controls. Therefore, the cases showed a protective phenotype from fatty liver, myosteatosis, and sarcopenia, which was quite an unexpected and new finding.

The mechanism of the metabolically protective phenotype of mild elevation of AFP is not known. In the adult liver, AFP expression is suppressed at the promoter and at two enhancers by corepressors (proteins or microRNA) and methylated histones. In HCC, hypermethylation and resultant silencing of repressors along with the microRNA-122 pathway may be mechanisms of AFP overexpression [31]. Recently, the nuclear receptor called liver receptor homolog-1 (LRH-1), also known as alpha-fetoprotein transcription factor (FTF) and NR5A2, was recognized as an attractive coordinator of hepatic metabolic function, including glucose and lipid processing, methyl group sensing, cellular stress responses, and bile acid and cholesterol homeostasis [32]. In particular, the LRH-1 agonist dilauroyl phosphatidylcholine reduces serum glucose levels and improves insulin sensitivity in db/db mice and high-fat diet-induced obese mice. Interestingly, LRH-1 controls the early expression of AFP [33], so unrevealed mechanisms regulating LRH-1 may elevate AFP levels mildly and play a role in expressing a metabolically protective phenotype. This may apply in selected patients without a history of chronic hepatitis such as chronic hepatitis B or C, as we have excluded them. However, this association should be confirmed by further studies revealing the detailed mechanism [34, 35].

To our knowledge, this study is the first to perform detailed body composition analysis using CT image in relation to AFP levels. However, there were some limitations to this study. First, this is a retrospective, single-center study. Due to the retrospective nature of this study, there was a lack of data on other acute phase reactants such as CRP or ferritin, which may affect AFP level. Also, information on physical activity or exercise was not available for analysis of muscle mass and fat. Second, CT analysis of body composition was limited because of the unavailability of pre-contrast CT images. We also analyzed the correlation by the Pearson method between AFP and the body composition by using only the study cohort with CT data, however, the results showed no significant correlation. This result may be due to the small number of patients enrolled in our study (S1 Fig). Third, patients with hemangiomas were used as the control group. However, hepatic hemangiomas are well-known benign lesions that do not cause AFP elevation, and CT was commonly performed for the evaluation of hemangiomas. Therefore, hemangioma patients were the only available patients closest to healthy controls. For reasonable comparison, we randomly selected the control group matched to age and sex, and large hemangioma patients were excluded because it could affect the hepatic fat measurement.

In conclusion, asymptomatic adults with elevated AFP levels may play a role in expressing a protective phenotype against hepatic steatosis, myosteatosis, and sarcopenia. Elevated AFP levels were stable without liver injury or cancer development. Further studies on the mechanism of elevated AFP levels in relation to fat metabolism are warranted in these asymptomatic individuals.

## Supporting information

S1 Table Baseline characteristics of the elevated AFP patients whose CT was analyzed in comparison with patients whose CT was not analyzed. Numerical variables are shown as median (IQR) except age, shown as mean ± SD. Categorical variables are shown as n (%). *Significant alcohol consumption: >30 g/day in male and >20 g/day in female. FIB-4 = (age x AST)/(platelets x √ALT). HSI = 8 x (ALT/AST ratio) + BMI (+2, if female; +2, if diabetes). NFS = -1.675 + 0.037 x age + 0.094 x BMI + 1.13 x diabetes + 0.99 x (AST/ALT ratio) - 0.013 x platelet—0.66 x albumin.
(DOCX)

S2 Table Variables associated with elevated AFP >7 ng/mL in patients without Gilbert’s syndrome. (DOCX)

S3 Table List of medication and herbal supplements. *Other medications included medication for cough, hair loss, headache, benign prostatic hyperplasia, ferrous sulfate and allergic rhinitis.
(DOCX)

S1 Fig Correlation between AFP and body composition in case group with CT image. (TIF)

## Abbreviations

AFP: alpha-fetoprotein
ALP: alkaline phosphatase
ALT: alanine aminotransferase
AST: aspartate aminotransferase
BMI: body mass index
FIB-4: fibrosis-4
γGT: gamma glutamyl peptidase
HSI: hepatic steatosis index
PT INR: prothrombin time international normalized ratio
NFS: nonalcoholic fatty liver disease fibrosis score
