# Supplementary material for: Elevated alpha-fetoprotein in asymptomatic adults: Clinical features, outcome, and association with body composition
Source: PLoS One. 2022 Jul 21;17(7):e0271407. doi: 10.1371/journal.pone.0271407 (PMC9302731; doi:10.1371/journal.pone.0271407)
Supplement: S1 Table — Numerical variables are shown as median (IQR) except age, shown as mean ± SD. Categorical variables are shown as n (%). *Significant alcohol consumption: >30 g/day in male and >20 g/day in female. FIB-4 = (age x AST)/(platelets x √ALT). HSI = 8 x (ALT/AST ratio) + BMI (+2, if female; +2, if diabetes). NFS = -1.675 + 0.037 x age + 0.094 x BMI + 1.13 x diabetes + 0.99 x (AST/ALT ratio) - 0.013 x platelet—0.66 x albumin. (DOCX) [file pone.0271407.s001.docx]

**Supplementary Table 1. Baseline characteristics of the elevated AFP patients whose CT was analyzed in comparison with patients whose CT was not analyzed.**

|  | **Total**  **(n=137)** | | **CT analyzed**  **(n=45)** | | **CT not analyzed**  **(n=92)** | | **p-value** |  |
| --- | --- | --- | --- | --- | --- | --- | --- | --- |
| Variables |  |  |  |  |  |  |  |  |
| Age, years | 47.5 ± 10.6 |  | 48.3 ± 11.3 |  | 48.3 ± 11.3 |  | 0.505 |  |
| Sex, male | 49 (35.8) |  | 21 (46.7) |  | 28 (30.4) |  | 0.095 |  |
| BMI, kg/m² | 22.2 (20.4 - 24.6) | (n=125) | 22.6 (21.0 -24.2) | (n=42) | 22.1 (20.2 - 24.8) | (n=83) | 0.415 |  |
| Obesity (BMI ≥25), % | 24 (19.2) |  | 6 (14.3) |  | 18 (21.7) |  | 0.452 |  |
| Overweight/Obesity (BMI ≥23), % | 50 (40.0) |  | 19 (45.2) |  | 31 (37.3) |  | 0.511 |  |
| Significant alcohol consumption* | 10 (8.5) | (n=118) | 4 (10.3) | (n=39) | 6 (7.6) | (n=79) | 0.891 |  |
| Alcohol intake, g/day | 3.4 (0.8 - 15.7) | (n=71) | 3.2 (1.9 - 15.7) | (n=23) | 4.0 (0.6 - 15.6) | (n=48) | 0.428 |  |
| Hypertension | 29 (21.2) |  | 12 (26.7) |  | 17 (18.5) |  | 0.379 |  |
| Diabetes | 6 (4.4) |  | 3 (6.7) |  | 3 (3.3) |  | 0.638 |  |
| Hyperlipidemia | 63 (46.0) |  | 20 (44.4) |  | 43 (46.7) |  | 0.944 |  |
| AFP, ng/dL | 10.3 (8.7 - 13.7) |  | 10.9 (9.6 - 13.9) |  | 10.3 (8.6 - 13.2) |  | 0.286 |  |
| WBC, x10^3^/µL | 5.5 (4.8 - 6.6) | (n=108) | 5.8 (4.8 -6.7) | (n=34) | 5.5 (4.8 - 6.5) | (n=74) | 0.294 |  |
| Hemoglobin, g/dL | 14.1 (13.2 - 15.0) | (n=108) | 14.1 (13.4 -15.2) | (n=34) | 13.9 (13.0 - 15.0) | (n=74) | 0.230 |  |
| Platelets, x10^9^/µL | 259.0 (224.0 - 292.0) | (n=109) | 237.5 (220.2 -285.2) | (n=34) | 263.0 (226.5 - 296.0) | (n=75) | 0.225 |  |
| Albumin, g/dL | 4.5 (4.3 - 4.7) | (n=120) | 4.5 (4.2 -4.7) | (n=41) | 4.5 (4.3 - 4.7) | (n=79) | 0.542 |  |
| Total bilirubin, mg/dL | 0.8 (0.6 - 1.0) | (n=122) | 0.7 (0.6 - 0.9) | (n=42) | 0.8 (0.6 - 1.0) | (n=80) | 0.226 |  |
| ALP, IU/L | 67.0 (57.2 - 79.0) | (n=118) | 66.5 (56.5 - 79.5) | (n=40) | 67.0 (58.2 - 78.0) | (n=78) | 0.805 |  |
| AST, IU/L | 21.0 (18.5 - 25.0) | (n=123) | 21.0 (19.0 - 24.0) | (n=42) | 21.0 (18.0 - 25.0) | (n=81) | 1.000 |  |
| ALT, IU/L | 17.0 (14.0 - 21.0) | (n=123) | 17.5 (14.0 - 21.0) | (n=42) | 17.0 (13.0 - 21.0) | (n=81) | 0.610 |  |
| γGT, IU/L | 18.0 (13.0 - 28.0) | (n=101) | 19.0 (13.0 - 24.0) | (n=35) | 18.0 (14.0 - 29.8) | (n=66) | 0.825 |  |
| Glucose, mg/dL | 95.0 (87.8 - 102.0) | (n=112) | 95.0 (87.0 - 103.0) | (n=37) | 95.0 (88.0 - 102.0) | (n=75) | 0.943 |  |
| Total cholesterol, mg/dL | 194.5 (172.2 - 210.0) | (n=122) | 188.5 (167.8 - 207.0) | (n=42) | 195.5 (177.5 - 214.2) | (n=80) | 0.243 |  |
| Hepatic steatosis index (HSI) | 34.1 (32.1 - 36.9) | (n=114) | 33.9 (31.7 - 36.8) | (n=40) | 34.5 (32.4 - 37.2) | (n=74) | 0.423 |  |
| HSI >36 | 37 (32.5) |  | 12 (30.0) |  | 25 (33.8) |  | 0.840 |  |
| NAFLD fibrosis score (NFS) | -2.8 (-3.4 - -2.1) | (n=97) | -2.6 (-3.3 - -1.9) | (n=32) | -2.9 (-3.4 - -2.2) | (n=65) | 0.195 |  |
| NFS grade |  |  |  |  |  |  | 0.200 |  |
| <-1.455 | 133 (95.9) |  | 42 (90.6) |  | 91 (98.5) |  |  |  |
| -1.455 - 0.676 | 4 (4.1) |  | 3 (9.4) |  | 1 (1.5) |  |  |  |
| >0.676 | 0 (0.0) |  | 0 (0.0) |  | 0 (0.0) |  |  |  |
| Fibrosis-4 index (FIB-4) | 1.0 (0.7 - 1.3) | (n=107) | 1.0 (0.8 - 1.3) | (n=33) | 0.9 (0.7 - 1.2) | (n=74) | 0.384 |  |
| FIB-4 grade |  |  |  |  |  |  | 0.961 |  |
| <1.3 | 83 (77.6) |  | 37 (75.8) |  | 76 (78.4) |  |  |  |
| 1.3 - 2.67 | 24 (22.4) |  | 8 (24.2) |  | 16 (21.6) |  |  |  |
| >2.67 | 0 (0.0) |  | 0 (0.0) |  | 0 (0.0) |  |  |  |
| Numerical variables are shown as median (IQR) except age, shown as mean ± SD. Categorical variables are shown as n (%).  *Significant alcohol consumption: >30 g/day in male and >20 g/day in female.  FIB-4=(age x AST)/(platelets x √ALT). HSI=8 x (ALT/AST ratio) + BMI (+2, if female; +2, if diabetes).  NFS=-1.675 + 0.037 x age + 0.094 x BMI + 1.13 x diabetes + 0.99 x (AST/ALT ratio) - 0.013 x platelet - 0.66 x albumin. | | | | | | | | |
|  | | | |  |  |  |  |  |
|  | | | | | | | |  |
|  | | | | | | | |  |
|  | | | |  |  |  |  |  |
|  | | | | | | | |  |
|  | | | | |  |  |  |  |
|  | | |  |  |  |  |  |  |
