# Supplementary material for: Elevated alpha-fetoprotein in asymptomatic adults: Clinical features, outcome, and association with body composition
Source: PLoS One. 2022 Jul 21;17(7):e0271407. doi: 10.1371/journal.pone.0271407 (PMC9302731; doi:10.1371/journal.pone.0271407)
Supplement: S2 Table — (DOCX) [file pone.0271407.s002.docx]

**Supplementary Table 2. Variables associated with elevated AFP >7 ng/mL in patients without Gilbert's syndrome.**

|  | **Univariate analysis** | | **Multivariate analysis** | |
| --- | --- | --- | --- | --- |
| **Variables** | **OR (95% CI)** | **p-value** | **OR (95% CI)** | **p-value** |
| Overweight/Obesity (BMI≥23kg/m^2^) | 0.594(0.380-0.902) | 0.026 | 0.520 (0.316-0.858) | 0.010 |
| Hypertension | 1.420 (0.085-2.373) | 0.181 |  |  |
| WBC, x10^3^/ µL | 0.933 (0.798-1.091) | 0.386 |  |  |
| Hemoglobin, g/dL | 1.048 (0.895-1.227) | 0.558 |  |  |
| Platelets, x10^9^/ µL | 0.999 (0.995-1.004) | 0.796 |  |  |
| Albumin, g/dL | 0.960 (0.418-2.203) | 0.923 |  |  |
| Total bilirubin, mg/dL | 5.703 (2.433-13.369) | <0.001 | 6.968 (2.792-17.388) | <0.001 |
| ALP, IU/L | 1.002 (0.991-1.013) | 0.775 |  |  |
| AST, IU/L | 0.999 (0.960-1.040) | 0.969 |  |  |
| ALT, IU/L | 0.979 (0.951-1.009) | 0.173 |  |  |
| γGT, IU/L | 0.998 (0.985-1.011) | 0.749 |  |  |
| Glucose, mg/dL | 0.995 (0.980-1.011) | 0.547 |  |  |
| Total cholesterol, mg/dL | 1.004 (0.998-1.011) | 0.178 |  |  |
| FIB-4 score | 1.284 (0.740-2.228) | 0.374 |  |  |
| NFS | 0.974 (0.769-1.234) | 0.827 |  |  |
| HSI | 0.971 (0.912-1.034) | 0.365 |  |  |
|  | | | | |
