# Supplementary material for: Elevated alpha-fetoprotein in asymptomatic adults: Clinical features, outcome, and association with body composition
Source: PLoS One. 2022 Jul 21;17(7):e0271407. doi: 10.1371/journal.pone.0271407 (PMC9302731; doi:10.1371/journal.pone.0271407)
Supplement: S3 Table — *Other medications included medication for cough, hair loss, headache, benign prostatic hyperplasia, ferrous sulfate and allergic rhinitis. (DOCX) [file pone.0271407.s003.docx]

| **Supplementary table 3. List of medication and herbal supplements.** | | | | | |  |  |  |  |  |
| --- | --- | --- | --- | --- | --- | --- | --- | --- | --- | --- |
|  | Total (n=411) | Case group (n=137) | Control group (n=274) | | p-value |  |  |  |  |  |
| Variables, No (%) |  |  |  |  |  |  |  |  |  |  |
| Medication | 122 (29.7) | 48 (35.0) | 74 (27.0) | | 0.093 |  |  |  |  |  |
| Hypertension | 37 (9.0) | 14 (10.2) | 23 (8.4) | | 0.542 |  |  |  |  |  |
| Diabetes mellitus | 10 (2.4) | 3 (2.2) | 7 (2.6) | | 1.000 |  |  |  |  |  |
| Dyslipidemia | 41 (10.0) | 22 (16.1) | 19 (6.9) | | 0.004 |  |  |  |  |  |
| Hypothyroidism | 11 (2.7) | 6 (4.4) | 5 (1.8) | | 0.191 |  |  |  |  |  |
| Omega-3 fatty acid | 13 (3.2) | 2 (1.5) | 11 (4.0) | | 0.235 |  |  |  |  |  |
| Aspirin | 5 (1.2) | 1 (0.7) | 4 (1.5) | | 0.669 |  |  |  |  |  |
| Others* | 66 (16.1) | 27 (19.7) | 39 (14.2) | | 0.154 |  |  |  |  |  |
| Herbal supplements | 70 (17.0) | 32 (23.4) | 38 (13.9) | | 0.016 |  |  |  |  |  |
| Multivitamin | 37 (9.0) | 16 (11.7) | 21 (7.7) | | 0.180 |  |  |  |  |  |
| Ginseng | 13 (3.2) | 7 (5.1) | 6 (2.2) | | 0.136 |  |  |  |  |  |
| Oriental herbal supplement | 7 (1.7) | 3 (2.2) | 4 (1.5) | | 0.690 |  |  |  |  |  |
| Herbal extracts | 12 (2.9) | 5 (3.6) | 7 (2.6) | | 0.544 |  |  |  |  |  |
| Lutein | 3 (0.7) | 1 (0.7) | 2 (0.7) | | 1.000 |  |  |  |  |  |
| *Other medications included medication for cough, hair loss, headache, benign prostatic hyperplasia, ferrous sulfate and allergic rhinitis. | | | | | |  |  |  |  |  |
|  | | | | | | | | | | |
|  | | | | | | |  |  |  |  |
|  | | | | | | | | | | |
|  | | | | | | | |  |  |  |
|  | | | |  | | |  |  |  |  |
