# Supplementary figures and images for: Elevated alpha-fetoprotein in asymptomatic adults: Clinical features, outcome, and association with body composition
Source: PLoS One. 2022 Jul 21;17(7):e0271407. doi: 10.1371/journal.pone.0271407 (PMC9302731; doi:10.1371/journal.pone.0271407)

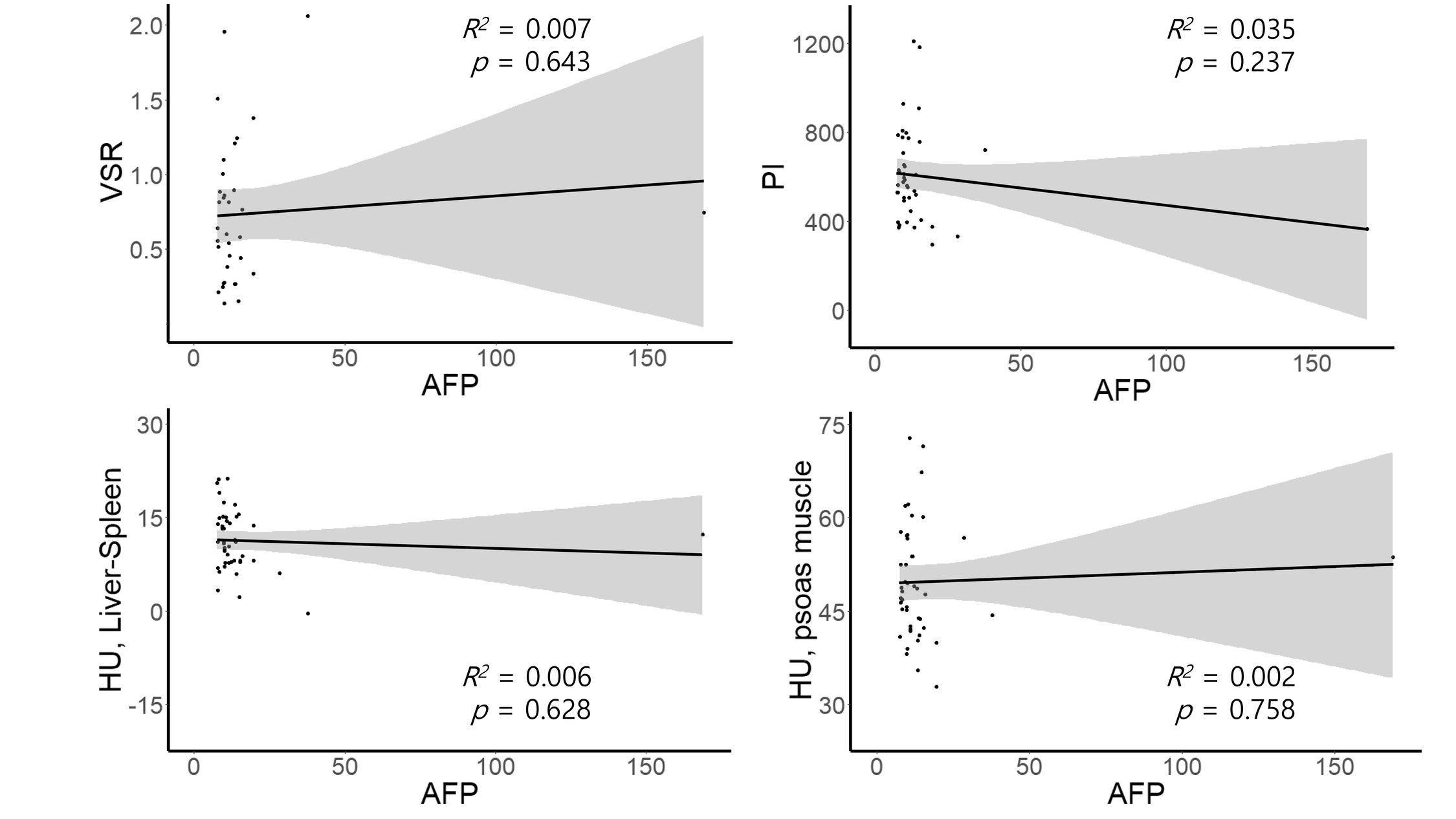

Supplement: S1 Fig — (TIF) [file pone.0271407.s004.tif]
